# Luteolin Limits Infarct Size and Improves Cardiac Function after Myocardium Ischemia/Reperfusion Injury in Diabetic Rats

**DOI:** 10.1371/journal.pone.0033491

**Published:** 2012-03-14

**Authors:** Dongdong Sun, Jie Huang, Zheng Zhang, Haokao Gao, Jiayi Li, Min Shen, Feng Cao, Haichang Wang

**Affiliations:** Department of Cardiology, Xijing Hospital, Fourth Military Medical University, Xi'an, Shaanxi, China; University of Padova, Medical School, Italy

## Abstract

**Background:**

*The* present study was to investigate the effects and mechanism of Luteolin on myocardial infarct size, cardiac function and cardiomyocyte apoptosis in diabetic rats with myocardial ischemia/reperfusion (I/R) injury.

**Methodology/Principal Findings:**

Diabetic rats underwent 30 minutes of ischemia followed by 3 h of reperfusion. Animals were pretreated with or without Luteolin before coronary artery ligation. The severity of myocardial I/R induced LDH release, arrhythmia, infarct size, cardiac function impairment, cardiomyocyte apoptosis were compared. Western blot analysis was performed to elucidate the target proteins of Luteolin. The inflammatory cytokine production were also examined in ischemic myocardium underwent I/R injury. Our results revealed that Luteolin administration significantly reduced LDH release, decreased the incidence of arrhythmia, attenuated myocardial infarct size, enhanced left ventricular ejection fraction and decreased myocardial apoptotic death compared with I/R group. Western blot analysis showed that Luteolin treatment up-regulated anti-apoptotic proteins FGFR2 and LIF expression, increased BAD phosphorylation while decreased the ratio of Bax to Bcl-2. Luteolin treatment also inhibited MPO expression and inflammatory cytokine production including IL-6, IL-1a and TNF-a. Moreover, co-administration of wortmannin and Luteolin abolished the beneficial effects of Luteolin.

**Conclusions/Significance:**

This study indicates that Luteolin preserves cardiac function, reduces infarct size and cardiomyocyte apoptotic rate after I/R injury in diabetic rats. Luteolin exerts its action by up-regulating of anti-apoptotic proteins FGFR2 and LIF expression, activating PI3K/Akt pathway while increasing BAD phosphorylation and decreasing ratio of Bax to Bcl-2.

## Introduction

The worldwide epidemic of diabetes mellitus is increasing the burden of cardiovascular disease, the leading cause of death among patients with diabetes [1]. Diabetes is now considered to be a risk equivalent of coronary artery disease for future MI and cardiovascular death [2]. Our previous study has shown that diabetes renders the heart more sensitive to I/R injury [3]. This warrants the significance of aggressive primary prevention against ischemia/reperfusion (I/R) injury in diabetic patients.

Diabetes is associated with significantly increased cardiomyocyte apoptosis [4], [5], [6], [7]. It is well documented that blocking the apoptosis process could prevent the loss of contractile cells, minimize cardiac I/R injury and therefore slow down the occurrence of heart failure [8]. FGFR2 and LIF are anti-apoptotic proteins which have been shown to be survival signal mediators in cardiomyocyte response against myocardial infarction [9], [10], [11]. Protective effects of LIF and FGFR2 were also related to up-regulation of the Akt Signaling [9], [10], [11]. Akt is known to regulate many survival pathways of the cardiac cells. Activation of Akt plays a pivotal role in fundamental cellular functions such as cell proliferation and survival by phosphorylating a variety of substrates. It has been reported that PI3K/Akt pathway regulates cardiac contractility and cardiomyocyte apoptosis [12]. Activation of PI3K/Akt pathway is an effective way to reduce cardiomyocyte apoptosis thus reduces cardiac I/R injury.

Luteolin, a flavonoid polyphenolic compound, is a widely distributed in many fruits and vegetables [13]. Studies in human beings as well as animal models have revealed the diverse beneficial effects of Luteolin, such as cardiovascular protection, antioxidant, anti-inflammatory, which suggest Luteolin is a valuable compound for many medical applications [14], [15], [16]. Luteolin has been shown to improve contractile function and attenuates apoptosis following I/R injury in adult rat cardiomyocytes [17]. In addition, Luteolin significantly enhanced left ventricular pressure and the global and relative coronary flow in Langendorff rabbit hearts subjected to repeated myocardial ischemia [18]. The potential effects of Luteolin on diabetes and I/R injury prompted us to investigate whether it is capable of exerting protection effects during cardiac I/R injury in diabetic rats and the underlying mechanism responsible for its effects.

Therefore, the aims of the present study were 1) to clarify whether Luteolin protects diabetic rats from cardiac I/R injury and cardiomyocytes apoptosis; 2) to identify the underlying mechanisms of Luteolin on I/R injury and cardiomyocytes apoptosis in diabetic rats.

## Methods

### Animals

The experiments were performed in adherence with the National Institutes of Health Guidelines on the Use of Laboratory Animals and were approved by the Fourth Military Medical University Ethic Committee on Animal Care (Approval ID: 2009055). One hundred and fifty adult male Sprague-Dawley (SD) rats, weight 200 to 220 g were purchased from the animal center in the Fourth Military Medical University. Diabetes mellitus (DM) was induced by intraperitoneal injections (i.p.) of STZ (50 mg/kg, STZ was dissolved in 0.1 M citrate buffer, pH 4.5) as previously described [19]. Rats were randomly allocated into the following groups with n = 30 each: (1) Non-DM+sham group (Non-DM); (2) DM+sham group (Sham)(Diabetic group); (3) DM+I/R group (I/R); (4) DM+Luteolin+I/R group (Luteolin); (5) DM+Luteolin+wortmannin group+I/R (Luteolin+W).

Blood glucose concentration was determined by using a reflectance meter (Accu-Chek, Roche Diagnostics GmbH, Mannheim, Germany) 1 week after STZ injection. Random blood glucose was tested in each rat for three times, all of these three values ≥16.7 mmol/L was considered as a cutoff point for diabetes. Blood glucose testing was also repeated at 3 and 8weeks after STZ injection. Eight weeks after STZ administration, Luteolin (10 µg/kg) (99%, Sigma, USA) was given by tail vein injection for three days. Wortmannin (a specific PI3K inhibitor, 16 µg/kg) was injected via the tail vein 5 min before Luteolin injection (Luteolin and Wortmannin were both dissolved in DMSO). Sham group received the same volume of DMSO for three days.

### LDH release evaluation

Myocardial cellular damage was evaluated by measuring lactate dehydrogenase (LDH) activity in plasma. LDH released from ischemic tissue was determined from arterial blood drawn from the carotid catheter 3 h after reperfusion. LDH activity was measured spectrophotometrically with a commercially available assay kit (Sigma, St Louis, MO, USA).

### Construction of I/R injury animal model and hemodynamic evaluation

I/R injury animal model was constructed by LAD ligation for 30 min followed by 3 h reperfusion eight weeks after STZ injection as previous described [3]. In brief, rats were anesthetized with 3% isoflurane. The chest was opened through a left thoracic incision. A 6–0 silk suture slipknot was placed at the distal 1/3 of the left anterior descending artery. The left ventricular pressure (LVP) was measured via a Millar Mikro-tip catheter transducer that was inserted into the left ventricular cavity through the left carotid artery. After 30 min of ischemia, the slipknot was released, and the myocardium was reperfused for 3 h. Sham operated control rats underwent the same surgical procedures except that the suture placed under the left coronary artery was not tied.

Cardiac function was continuously determined by invasive hemodynamic evaluation methods during the entire I/R period. The first derivative of the left ventricular pressure (+dp/dt max and −dp/dt max), heart rate (HR), blood pressure (BP), and Electrocardiogram (ECG) were obtained by use of computer algorithms and an interactive videographics program (Po-Ne-Mah Physiology Platform P3 Plus, Gould Instrument Systems, Valley View, Ohio). Premature ventricular beats (PVB), ventricular tachycardia (VT) and ventricular fibrillation (VF) were evaluated according to the diagnostic criteria advocated by the Lambeth Convention [20].

### Measurement of Myocardial Infarct Size

Myocardial Infarct Size was evaluated by Evans Blue/TTC staining as previously described [3]. Three hours after reperfusion, the ligature around the coronary artery was retied, and 1 ml of 2% Evans Blue dye was injected into the side arm of the aortic cannula. The heart was quickly excised after the dye was uniformly distributed, frozen at −80°C and sliced transversally into 1 mm thick sections. The slices were incubated in 1% 2, 3, 5-triphenyltetrazolium chloride (TTC, Sigma-Aldrich, St Louis, Mo) for 10 min at 37°C. Evans blue stained areas indicated area-not-at-risk (ANAR). Red parts in the heart, which were stained by TTC, represented for ischemic but viable tissue. Staining negative areas indicated infarcted myocardium. Areas of infarct size (IS) and area-at-risk (AAR) were measured digitally using Image Pro Plus software (Media Cybernetics). IS and AAR were expressed as percentages of the left ventricular area (IS/LV and AAR/LV respectively).

### Determination of Myocardial Apoptosis

Myocardial apoptosis was determined by terminal deoxynucleotidyl transferase-mediated dUTP-biotin nick end labeling (TUNEL) staining and caspase 3 activity assay as previously described [3]. TUNEL staining was performed with fluorescein-dUTP (In Situ Cell Death Detection Kit; Roche Diagnostics) for apoptotic cell nuclei and 4′,6-diamidino-2-phenylindole (DAPI) (Sigma) stained all cell nuclei. Additional staining was performed using a monoclonal antibody against Troponin I (cTnI, Santa Cruz) for the identification of myocardium. Apoptotic index (AI) was determined. AI = number of TUNEL-positive myocytes/total number of myocytes stained with DAPI from a total of 40 fields per heart (n = 5). All of these assays were performed in a blinded manner. Caspase-3 activity was measured with the ApoAlert Caspase-3 Assay Plate (Clontech, Mountain View, Calif) according to the manufacturer's instructions [21]. Substrate cleavage was monitored fluorometrically with a SpectraMax Gemini spectrophotometer (Molecular Devices) with excitation and emission wavelengths of 350 and 450 nm.

### Determination of Cardiac Function

Echocardiography was conducted at 24 h after infarction as previously described [19]. Sedated rats (3% isoflurane) were studied on an echocardiography system (Sequoia Acuson, Siemens; 15-MHz linear transducer). Cardiac dimensions and function were assessed by M-mode echocardiography. Left ventricular end-diastolic diameter (LVEDD) and Left ventricular end-systolic diameter (LVESD) were measured on the parasternal left ventricular long axis view. All measurements represent the mean of 5 consecutive cardiac cycles. Left ventricular end-systolic volume (LVESV), Left ventricular end-diastolic volume (LVEDV) and Left ventricular ejection fraction (LVEF) were calculated by use of computer algorithms. All of these measurements were performed in a blinded manner.

### Western blot evaluation

Protein was isolated from homogenized heart tissue with Trizol reagent (Invitrogen, Carlsbad, Calif) and standard Invitrogen protocols. After protein concentration quantitation with the modified Bradford assay (Bio-Rad Laboratories, Hercules, Calif), protein was then used for Western blotting with primary antibodies against caspase-3, cleaved caspase-3, FGFR2, LIF, Akt, Akt-P (Thr 308, Ser 473), BAD, BAD-P (Ser 136), Bax, Bcl-2 (Santa Cruz Biotechnology, Santa Cruz, Calif). The blots were visualized with a chemiluminescene system (Amersham Bioscience, Buchinghamshire, UK). The signals were quantified by Image Pro Plus software (Media Cybernetics).

### Determination of tissue myeloperoxidase (MPO), interleukin-6 (IL-6), IL-1α and tumor necrosis factor-alpha (TNF-α) activity

Following the 3 h reperfusion period, tissue sample were taken from the AAR zones for MPO activity analysis. The activity of MPO was measured spectrophotometrically at 460 nm and expressed as units per 100 mg of tissue. The concentrations of IL-6, IL-1α and TNF-α were measured by enzyme-linked immunosorbent assay (ELISA) according to the manufacturer's instructions as previously described [3]. Values are expressed as pg/mg of total protein.

### Statistical analysis

Continuous variables that approximated the normal distribution were expressed as means ± SD. Comparison between groups were subjected to ANOVA followed by Bonferroni correction for post hoc t-test. Data expressed as proportions were assessed with a Chi-square test. Two sided tests have been used throughout, and *P* values<0.05 were considered statistically significant. SPSS software package version 14.0 (SPSS, Chicago, IL) was used for data analysis.

## Results

### Baseline parameters

No major differences were found between groups in terms of heart rate and blood pressure before the I/R injury. Blood glucose and the heart to body mass ratio were significantly higher in the diabetic group as compared with the non-diabetic group. Body mass were lower in the diabetic group as compared with the non-diabetic group (Table 1).

**Table 1:** Basic Parameters of the Rats.

Basic Parameters	Non-DM	Sham	I/R	Luteolin	Luteolin+W	P
	(n = 30)	(n = 30)	(n = 30)	(n = 30)	(n = 30)	
Heart rate (min^−1^)	430.1±21.7	432.5±22.6	427.1±19.8	435.9±24.4	423.7±18.2	0.47
Blood glucose (mmol/L)	4.9±0.5	22.6±2.7	23.1±1.4	22.1±2.5	24.3±2.7	<0.001
Blood pressure (mmHg)	82.3±3.9	83.2±4.6	80.9±3.5	85.6±4.7	84.9±2.8	0.39
Body mass (g)	443.9±42.6	406.5±36.7	398.2±28.5	412.8±35.9	409.1±33.2	<0.001
Heart to bodymass ratio (mg/g)	1.96±0.19	2.17±0.12	2.23±0.18	2.21±0.25	2.14±0.28	<0.001

Values are presented as mean ± SD.

### Luteolin reduces arrhythmia and LDH release after I/R injury in diabetic rats

**Figure 1:**
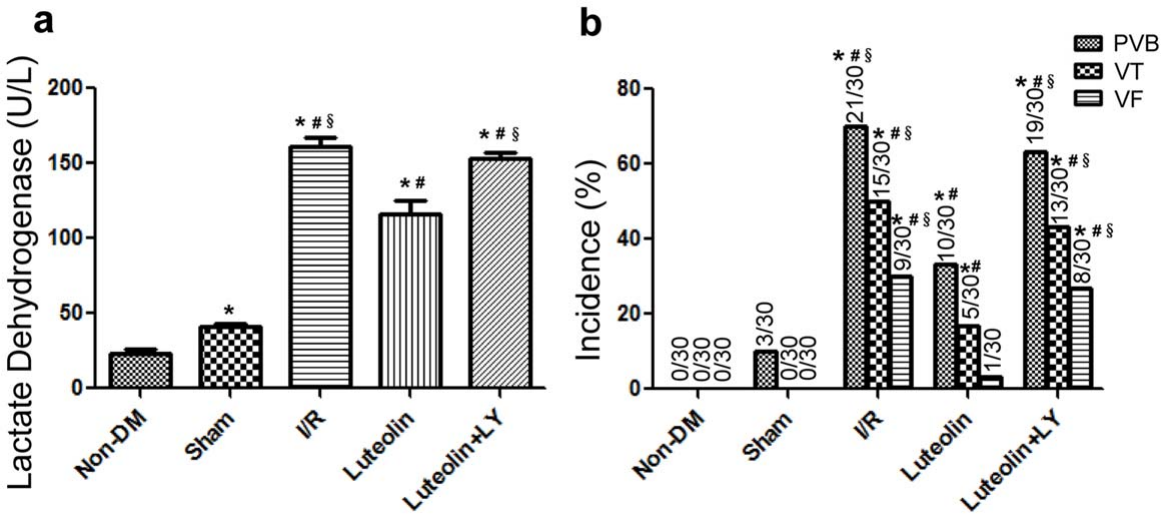
Luteolin reduces LDH release and I/R induced arrhythmia in diabetic rats. Diabetes increased LDH release compared with the non-diabetic group. Luteolin reduced the LDH release after I/R injury in diabetic mice. The PI3K inhibitor wortmannin pretreatment statistically increased the release of LDH compared with the Luteolin group (a). Luteolin decreased the incidence of PVB, VT and VF attacks as compared with the I/R group. Wortmannin abolished the anti-arrhythmia effects of Luteolin (b). PVB: premature ventricular beats; VT: ventricular tachycardia; VF, ventricular fibrillation. The columns and errors bars represent means and SD. * *p*<0.05 *vs* Non-DM, ^#^ *p*<0.05 *vs* Sham, **^§^** *p*<0.05 *vs* Luteolin.

Luteolin administration significantly decreased the release of lactate dehydrogenase (LDH), a biochemical marker for necrotic cell death, as compared with the I/R group (116.6±19.9 *vs* 161.6±12.1, *P* = 0.003). However, the PI3K inhibitor wortmannin pretreatment statistically increased the release of LDH compared with the Luteolin group (153.4±8.9 *vs* 116.6±19.9, *P* = 0.005) (Figure 1a). I/R injury induced frequent attacks of premature ventricular beats (PVB), ventricular tachycardia (VT) and ventricular fibrillation (VF). Luteolin significantly decreased the incidence of PVB (33.3% *vs* 70%, *P* = 0.009), VT (16.7% *vs* 50%, *P* = 0.013) and VF (3.3% *vs* 30%, *P* = 0.012) as compared with the I/R group. Moreover, wortmannin abolished the anti-arrhythmia effects of Luteolin (PVB: 63.3% *vs* 33.3%, *P* = 0.038; VT 43.3% *vs* 16.7%, *P* = 0.047; VF 26.7% *vs* 3.3%, *P* = 0.026) as compared with the Luteolin group (Figure 1b).

### Luteolin decreases infarct size after I/R injury in diabetic rats

**Figure 2:**
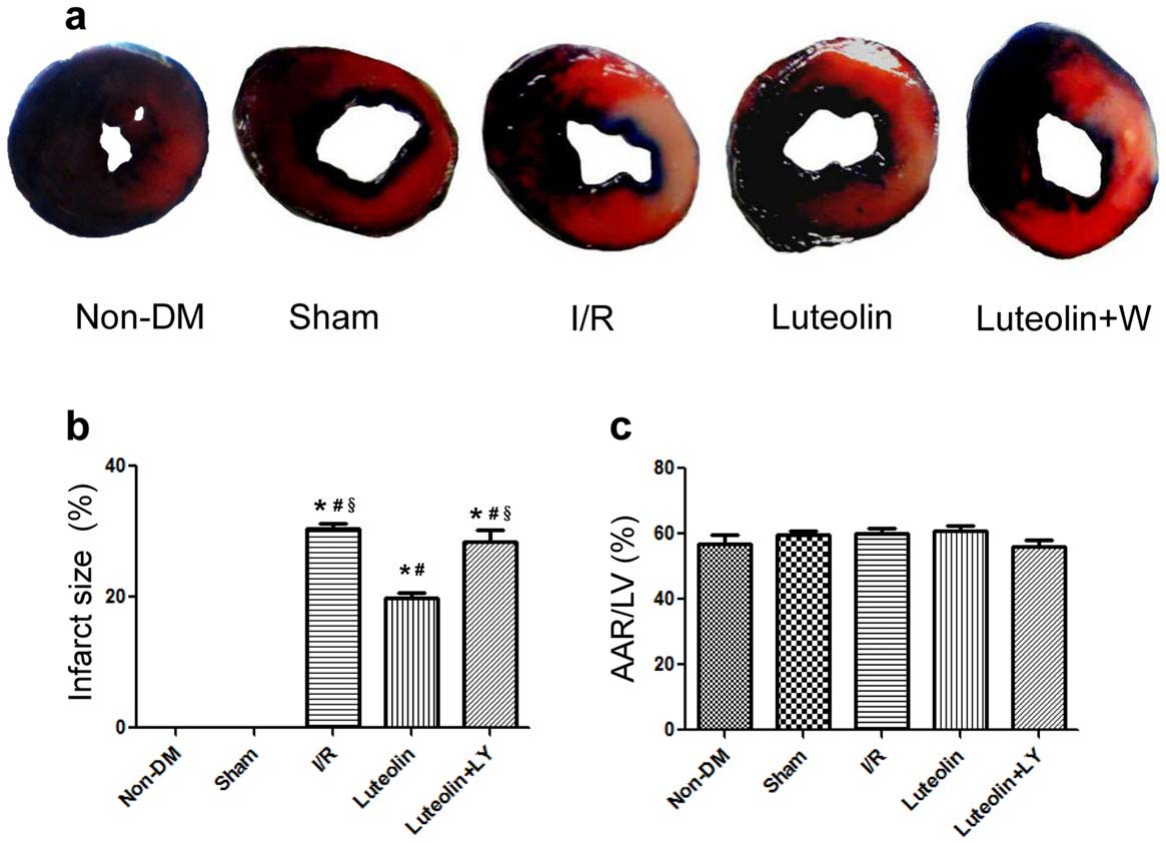
Luteolin decreases infarct size in diabetic rats subjected to I/R injury. Representative illustrations of infarct size as stained by Evans Blue and TTC (a). Luteolin decreased infarct size 3 h after I/R injury compared with the I/R group and the wortmannin group (b). The risk of area had no statistical difference between the groups (c). The columns and errors bars represent means and SD. * *p*<0.05 *vs* Non-DM, ^#^ *p*<0.05 *vs* Sham, **^§^** *p*<0.05 *vs* Luteolin.

Representative images of infarct size as stained by Evans Blue and TTC were shown in Figure 2a. Luteolin administration significantly decreased infarct size at 3 h (0.199±0.016 *vs* 0.304±0.017, *P<*0.001) after I/R injury compared with the I/R group. This effect was abolished by wortmannin treatment (0.285±0.038 in wortmannin group *vs* 0.199±0.016 in Luteolin group, *P* = 0.002) (Figure 2b). No significant difference in risk area was found between the four groups (Figure 2c).

### Luteolin enhances left ventricular function after I/R injury

**Figure 3:**
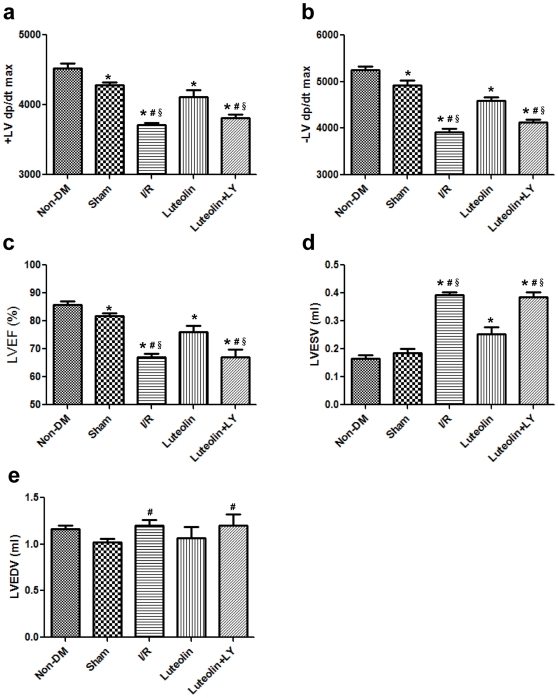
Luteolin enhances left ventricular function evaluated by hemodynamic measurements and echocardiograpy. Hemodynamic measurements indicated that diabetes decreased the ± LV dp/dt max compared with the non-diabetic group. I/R induced significant decrease of ± LV dp/dt max in diabetic rats. Luteolin enhanced the ± LV dp/dt max compared with the I/R group. The PI3K inhibitor wortmannin abolished the effects of Luteolin on the ± LV dp/dt max (a, b). Luteolin administration significantly enhanced LVEF as compared with the I/R group and the wortmannin group (c). Luteolin significantly inhibited the increase of LVESV and LVEDV compared with the I/R group and the wortmannin group (d, e). LVEF: Left ventricular ejection fraction; LVESV: Left ventricular end-systolic volume; LVEDV: Left ventricular end-diastolic volume. The columns and errors bars represent means and SD. * *p*<0.05 *vs* Non-DM, ^#^ *p*<0.05 *vs* Sham, **^§^** *p*<0.05 *vs* Luteolin.

Hemodynamic measurements were performed 3 h after I/R injury (Figure 3a, 3b). Diabetes significantly decreased the ±LV dp/dt max compared with the non-diabetic group (Figure 3a, 3b). The +LV dp/dt max (3710.2±72.5 *vs* 4284.6±90.6 mmHg/s, *P*<0.001) and the −LV dp/dt max (3920.4±152.2 *vs* 4920.8±231.2 mmHg/s, *P*<0.001) were decreased after I/R injury in diabetic rats compared with the sham group. Luteolin significantly enhanced the +LV dp/dt max (4115.0±210.3 *vs* 3710.2±72.5 mmHg/s, *P* = 0.004) and the −LV dp/dt max (4592.8±150.5 *vs* 3920.4±152.2 mmHg/s, *P*<0.001) compared with the I/R group. The PI3K inhibitor wortmannin abolished the effects of Luteolin on the +LV dp/dt max (3813.2±101.1 *vs* 4115.0±210.3 mmHg/s, *P* = 0.020) and the −LV dp/dt max (4123.8±155.0 *vs* 4592.8±150.5 mmHg/s, *P* = 0.001).

LVEF, ESV, EDV were evaluated by echocardiography 24 h after I/R injury (Figure 3c, 3d, 3e). Diabetes decreased LVEF compared with the non-diabetic group (Figure 3c). Luteolin administration significantly enhanced LVEF as compared with the I/R group (0.761±0.047 *vs* 0.671±0.028, *P* = 0.006) and the wortmannin group (0.761±0.047 *vs* 0.669±0.063, *P* = 0.031). Luteolin significantly inhibited the increase of LVESV compared with the I/R group (0.252±0.058 *vs* 0.394±0.018 ml, *P*<0.001) and the wortmannin group (0.252±0.058 *vs* 0.386±0.036 ml, *P* = 0.002). Luteolin also decreased LVEDV changes as compared with the I/R group (1.07±0.26 *vs* 1.20±0.13 ml, *P* = 0.046) and the wortmannin group (1.07±0.26 *vs* 1.20±0.24 ml, *P* = 0.045).

### Luteolin increases antiapoptotic protein expression and inhibites cardiomyocytes apoptosis after I/R injury in diabetic rats

**Figure 4:**
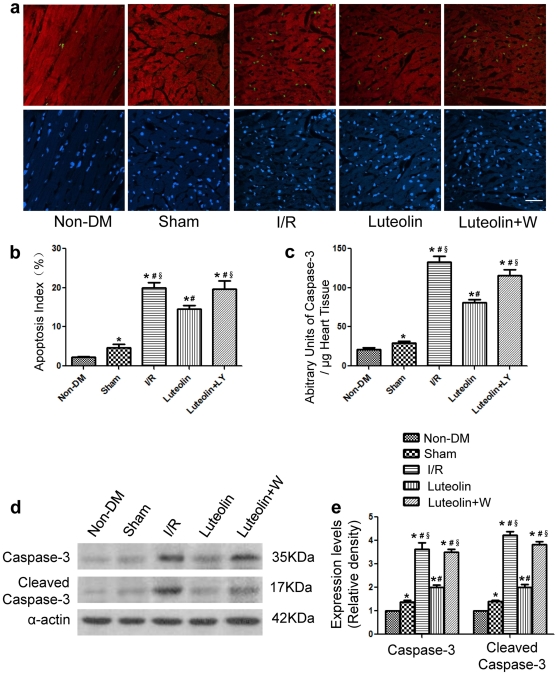
Luteolin decreases cardiomyocytes apoptosis in diabetic rats with I/R Injury. Representative photomicrograph showed that TUNEL-positive cardiomyocytes were more frequently observed in the I/R group and the wortmannin group compared with the Luteolin group (a). The apoptotic rate was higher in the diabetic group compared with the diabetic group. The number of TUNEL-positive cardiomyocytes (in green, DAPI in blue) was significantly reduced in the Luteolin group than in the I/R group and the wortmannin group (b). Luteolin treatment also significantly decreased caspase-3 activity compared with the I/R group and the wortmannin group (c). Luteolin decreased caspase-3 and cleaved caspase-3 expression as compared with the I/R group and the wortmannin group (d, e).Scale bar: 25 ìm. The columns and errors bars represent means and SD. * *p*<0.05 *vs* Non-DM, ^#^ *p*<0.05 *vs* Sham, **^§^** *p*<0.05 *vs* Luteolin.

The apoptotic rates were significantly higher in the diabetic group compared with the non-diabetic group. Diabetes enhanced caspase-3 activity and caspase-3 expression compared with the non-diabetic group (Figure 4). Representative photomicrograph showed that TUNEL-positive cardiomyocytes were more frequently observed in the I/R group and the wortmannin group as compared with the Luteolin group (Figure 4a). Quantitative analyses demonstrated that the number of TUNEL-positive cardiomyocytes was significantly less in the Luteolin group than in the I/R group (0.146±0.018 *vs* 0.20±0.03, *P* = 0.009) and the wortmannin group (0.146±0.018 *vs* 0.197±0.047, *P* = 0.032) (Figure 4b). Concurrently, Luteolin group showed significantly decreased caspase-3 enzymatic activity compared with the I/R group (80.8±8.3 *vs* 133.2±15.3, *P*<0.001) and the wortmannin group (80.8±8.3 *vs* 115.4±16.5, *P* = 0.003) (Figure 4c). Western blot analysis also demonstrated decreased caspase-3 and cleaved caspase-3 expression in the Luteolin group as compared with the I/R group and the wortmannin group (Figure 4d, 4e).

**Figure 5:**
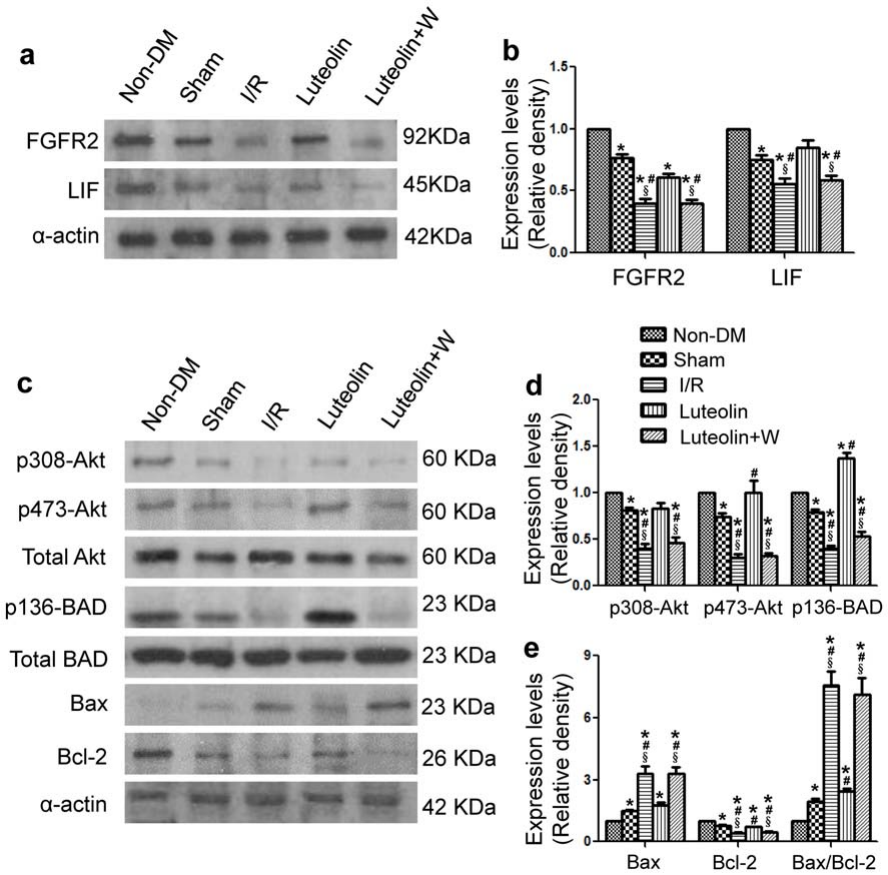
Western blot analysis. Western blot analysis revealed that Luteolin treatment increased the expression of antiapoptotic proteins FGFR2 and LIF as compared with the I/R group and wortmannin group (a, b). Luteolin also enhanced phosphorylation of Akt and BAD, increased Bax expression while decreased Bcl-2 expression resulted in decreased Bax/Bcl-2 ratio in cardiac tissue that were exposed to I/R injury (c, d, e). The PI3K inhibitor wortmannin abolished the effects of Luteolin on antiapoptotic proteins expression. The columns and errors bars represent means and SD. * *p*<0.05 *vs* Non-DM, ^#^ *p*<0.05 *vs* Sham, **^§^** *p*<0.05 *vs* Luteolin.

Western blot analysis revealed that the expression of anti-apoptotic proteins FGFR2 and LIF were decreased in the diabetic group as compared with the non-diabetic group. Diabetes decreased p308-Akt, p473-Akt, p136-BAD, Bcl-2 expression and Bax/Bcl-2 ratio, increased the expression of Bax as compared with the non-diabetic group (Figure 5). After 3 h of reperfusion, Luteolin treatment was associated with a significant increase in the expression of FGFR2 and LIF as compared with the I/R group (Figure 5a, 5b). Luteolin pretreatment also enhanced phosphorylation of Akt and BAD, decreased Bax expression while increased Bcl-2 expression resulted in decreased Bax/Bcl-2 ratio in cardiac tissue that were exposed to I/R injury (5c, 5d, 5e). Interestingly, the PI3K inhibitor wortmannin abolished the effects of Luteolin on anti-apoptotic proteins expression. Wortmannin administration was associated with reduced expression of FGFR2, LIF, p308-Akt, p473-Akt, Bcl-2 and increased expression of p136-BAD and Bax as indicated in Figure 5.

### Luteolin treatment alleviates leukocyte infiltration and reduces cytokine levels after I/R injury in diabetic rats

**Figure 6:**
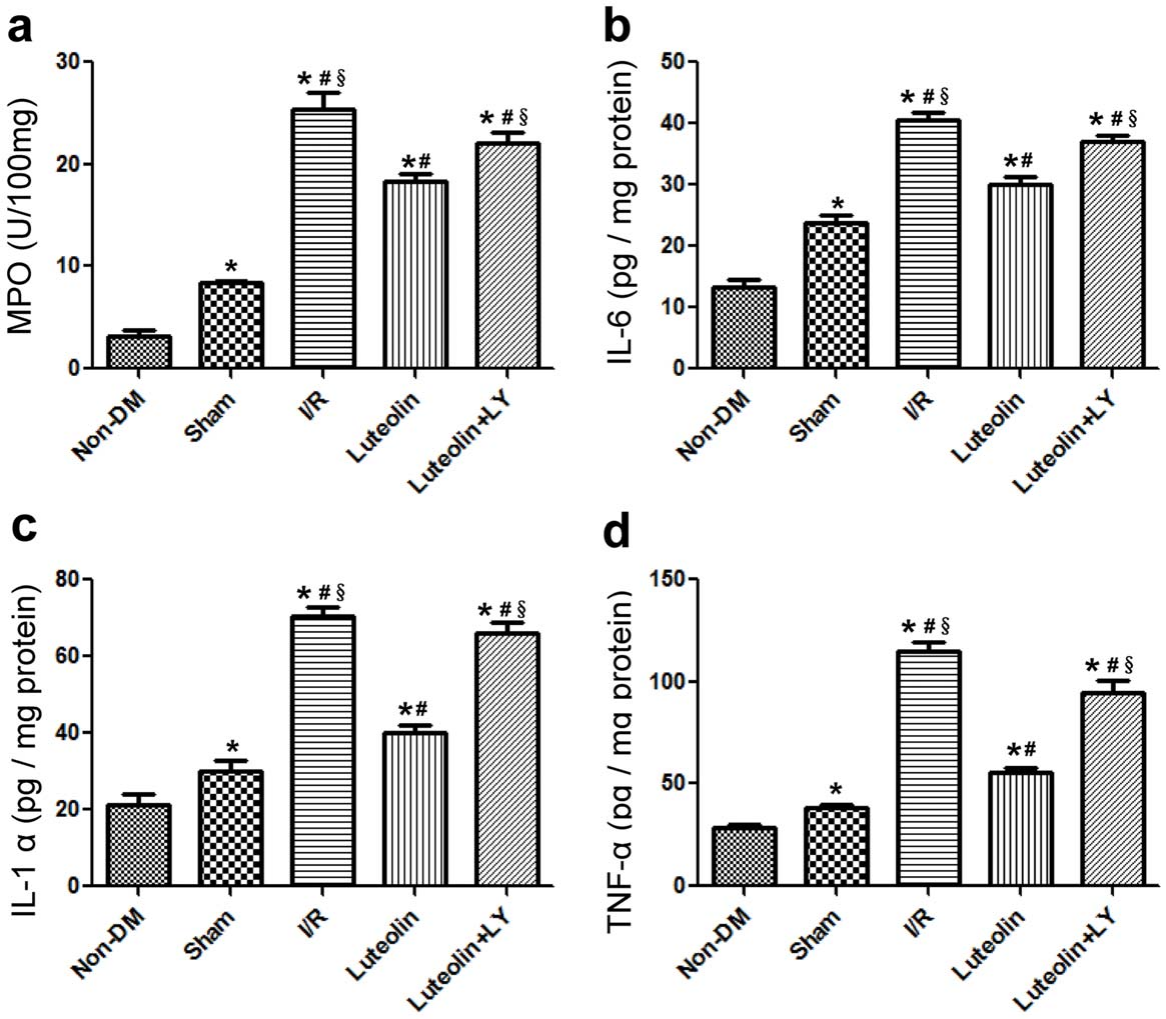
Luteolin alleviates leukocyte infiltration and reduces cytokine levels after I/R injury in diabetic rats. Luteolin reduced the MPO activity compared with the I/R group and the wortmannin group (a). Luteolin reduced the levels of IL-6, IL-1α and TNF-α production compared with the I/R group and the wortmannin group (b, c, d). The columns and errors bars represent means and SD. * *p*<0.05 *vs* Non-DM, ^#^ *p*<0.05 *vs* Sham, **^§^** *p*<0.05 *vs* Luteolin.

MPO activity was increased in the diabetic group as compared with the non-diabetic group. Following 3 h of reperfusion, the activity of MPO was significantly elevated in the I/R group when compared to the sham group (25.4±3.5 *vs* 8.4±0.6 U/100 mg, *P*<0.001). Treatment with Luteolin reduced the MPO activity compared with the I/R group (18.3±1.6 *vs* 25.4±3.5 U/100 mg, *P* = 0.003) and the wortmannin group (18.3±1.6 *vs* 22.1±2.3 U/100 mg, *P* = 0.016) (Figure 6a).

Elisa analysis demonstrated that diabetes increased the levels of left ventricular IL-6, IL-1α and tumor necrosis factor-α (TNF-α) as compared with the non-diabetic group (Figure 6b, 6c, 6d). I/R resulted in a noticeable increase in IL-6 (40.6±2.5 vs 23.9±2.8 pg/mg protein, *P*<0.001), IL-1α (70.4±5.9 vs 30.0±6.1 pg/mg protein, *P*<0.001) and TNF-α (115.1±9.0 vs 38.6±2.8 pg/mg protein, *P*<0.001) compared with the sham group. Luteolin reduced the levels of IL-6 (29.9±3.3 vs 40.6±2.5 pg/mg protein, *P*<0.001), IL-1α (40.2±4.4 vs 70.4±5.9 pg/mg protein, *P*<0.001) and TNF-α (55.9±4.8 vs 115.1±9.0 pg/mg protein, *P*<0.001) compared with the I/R group. Moreover, wortmannin administration significantly increased IL-6 (37.1±2.1 vs 29.9±3.3 pg/mg protein, *P* = 0.003), IL-1α (66.0±6.5 vs 40.2±4.4 pg/mg protein, *P*<0.001) and TNF-α (94.7±13.4 vs 55.9±4.8 pg/mg protein, *P*<0.001) production compared with the Luteolin group.

## Discussion

Coronary artery disease is associated with a less favorable outcome in diabetic than in non-diabetic patients. Accumulating evidence indicates that diabetes is associated with 2–4 times increased risk of CHD mortality compared with patients without diabetes [3], [22]. The flavone Luteolin, is widely distributed in many fruits, vegetables and traditional Chinese herbs [14]. Several pieces of evidences have shown that Luteolin is capable of protecting the myocardium against IR injury [23]. Clinical observations showed that regular Luteolin intake was associated with a reduced risk of cardiovascular diseases [24]. The beneficial effects were further supported by experimental studies in rabbit or guinea pig hearts showing enhancement of left ventricular pressure and coronary flow by Luteolin with or without regional ischemia [18], [25]. However, the direct cardio-protective effects of Luteolin on I/R injury in diabetic rats and the exact mechanism of its therapeutic action are still poorly understood. This promoted an investigation of the protective effects of Luteolin on I/R injury in diabetic rats and the underlying mechanism.

The present study has provided the evidence that diabetes decreased ± LV dp/dt max, LVEF and caspase-3 expression, while increased LDH release and cardiomyocyte apoptotic rate.

Luteolin reduced incidence of arrhythmia, reduced LDH release and decreased infarct size of diabetic rats subjected to I/R injury. We also demonstrated that Luteolin significantly improved the left ventricular function via increasing ± dp/dt max, LVEF and limiting the increase of LVESV and LVEDV. Similar to our results, Fang et al [17], [26] reported that Luteolin inhibits apoptosis and improves cardiomyocyte contractile function in Langendorff perfused rat hearts and isolated cardiomyocyte subjected to simulated I/R injury.

Cardiomyocyte apoptosis is one of the major contributors to the development of heart failure after myocardial infarction. Blocking the apoptosis process could prevent the loss of contractile cells, minimize cardiac injury induced by I/R injury and therefore slow down the occurrence of heart failure [8], [27]. Given the markedly improved recovery of left ventricular function exerted by Luteolin, we determined the effects of Luteolin on post-ischemic cellular damage. The extent of necrotic and apoptotic cell death was examined. The release of lactate dehydrogenase (LDH), a biochemical marker for necrotic cell death, was significantly decreased in the Luteolin treatment group. Luteolin administration significantly decreased TUNEL-positive cardiomyocytes and reduced caspase-3 expression in diabetic rats underwent I/R injury.

Both FGFR2 and LIF are anti-apoptotic proteins which have been shown to reduce cardiomyocyte apoptosis in myocardial I/R injury [28]. Most studies suggest that the Bcl-2 family is key regulators of physiological and pathological apoptosis. The family consists of both cell death promoters such as Bax, Bad and cell death inhibitors, which include Bcl-2, Bcl-X, etc. It has been demonstrated that the high ratio of Bax/Bcl-2 is associated with greater vulnerability to apoptotic activation [29], [30]. In the present study, we confirmed that Luteolin may exert its anti-apoptotic effects through up-regulating FGFR2, LIF expression, increasing BAD phosphorylation and Bcl-2 expression while down-regulating Bax expression and decreasing the ratio of Bax to Bcl-2.

It has been reported that PI3K/Akt pathway activation may lead to BAD phosphorylation and may thereby suppress cell apoptosis [28]. More interesting, protective effects of LIF and FGFR2 were also related to up-regulation of the Akt Signaling [9], [10], [11]. Hence, we next examined whether the Akt signaling was activated in diabetic rat hearts subjected to I/R injury. The specific PI3K inhibitor wortmannin was employed to observe the effects of co-administration of wortmannin and Luteolin compared with Luteolin alone. Co-administration of wortmannin and Luteolin increased infarct size, worsened left ventricular function, enhanced cardiomyocytes apoptosis, decreased anti-apoptotic proteins FGFR2 and LIF expression, and associated with increased ratio of Bax to Bcl-2. These results indicated that wortmannin can abolish the cardiac protective effects of Luteolin. The cardio-protective and anti-apoptotic effects of Luteolin were associated with, at least in part, the activation of PI3K/Akt-pathway.

Proinflammatory cytokines such as tumor necrosis factor-α (TNF-α), IL-6 and IL-1α have emerged as significant contributors to myocardial dysfunction [31]. Xagorari reported that Luteolin effectively inhibited the lipopolysaccharide-induced TNF-a and IL-6 production in an activated macrophage-like cell line [32]. In the present study, we also found that Luteolin inhibited inflammatory cytokine production including IL-6, IL-1α and TNF-a. Extracellular MPO can be determined as an index of polymorphonuclear leukocyte infiltration in response to inflammation. Luteolin administration significantly decreased MPO expression compared with the I/R group. Co-administration of wortmannin and Luteolin could also increase the expression of MPO, IL-6, IL-1α and TNF-a indicating that wortmannin abolishes the anti-inflammatory effects of Luteolin.

In conclusion, the salient finding of the present study is that Luteolin pretreatment reduces infarct size and improves cardiac hemodynamics after I/R injury in diabetic rats. This was through decreased cardiac apoptosis and inflammation. Luteolin exerted its protective effects by up-regulating of anti-apoptotic proteins FGFR2 and LIF expression, which activated PI3K/Akt pathway and associated with increased BAD phosphorylation and decreased ratio of Bax to Bcl-2. The present study may provide important insights for the understanding of the molecular mechanisms involved in the cardioprotective effect of Luteolin in diabetic rats underwent I/R injury.
